# Predicting the One-Particle Density Matrix with Machine Learning

**DOI:** 10.1021/acs.jctc.4c00042

**Published:** 2024-05-31

**Authors:** S. Hazra, U. Patil, S. Sanvito

**Affiliations:** School of Physics and CRANN Institute, Trinity College, Dublin 2, Ireland

## Abstract

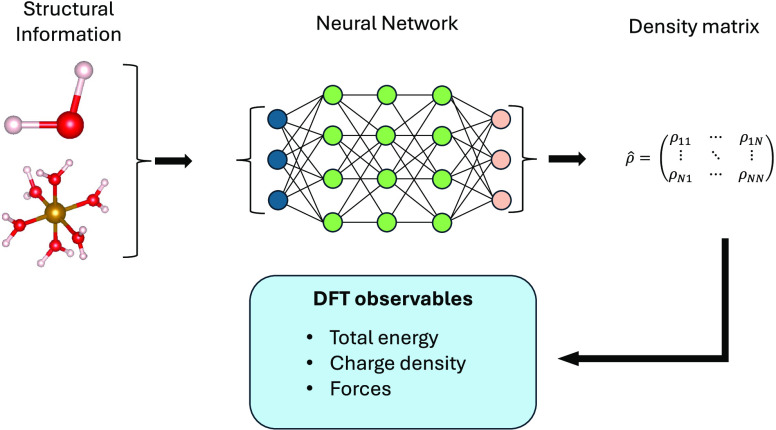

Two of the most widely used electronic-structure theory methods, namely, Hartree–Fock and Kohn–Sham density functional theory, require the iterative solution of a set of Schrödinger-like equations. The speed of convergence of such a process depends on the complexity of the system under investigation, the self-consistent-field algorithm employed, and the initial guess for the density matrix. An initial density matrix close to the ground-state matrix will effectively allow one to cut out many of the self-consistent steps necessary to achieve convergence. Here, we predict the density matrix of Kohn–Sham density functional theory by constructing a neural network that uses only the atomic positions as information. Such a neural network provides an initial guess for the density matrix far superior to that of any other recipes available. Furthermore, the quality of such a neural-network density matrix is good enough for the evaluation of interatomic forces. This allows us to run accelerated ab initio molecular dynamics with little to no self-consistent steps.

## 1 Introduction

Density functional theory (DFT)^1−3^ has been playing a central role in the electronic structure calculations of molecules and solids for more than six decades. The DFT success boils down to the rigorous theoretical framework,^1^ the availability of well-controlled approximations of the exchange–correlation functional,^4^ the multitude of numerical implementations,^5−11^ and the community rigor in benchmarking results.^12,13^ In principle, for a given functional, one can find the ground-state density and energy by functional minimization with respect to the electron density, a procedure denoted as orbital-free DFT.^14−16^ However, the lack of a universal and accurate approximation to a functional form of the noninteracting kinetic energy, a shortfall hardly mitigated by machine learning (ML),^17^ makes the widespread use of orbital-free DFT impractical. The problem can be circumvented by the Kohn–Sham (KS) construct,^2^ in which the minimization of the functional is performed by solving an associated system of single-particle Schrödinger-like equations. The one-particle potential entering the KS equations does, in turn, depend on the electron density. Hence, the solution is iterative and requires a multistep cycle, where the electron density and the KS potential are continuously updated until convergence is reached. Such a self-consistent field (SCF) process is common to other electronic structure methods, for instance, the Hartree–Fock scheme, where one updates the coefficients of the molecular orbitals.^18^

In general, the number of iterations required by the SCF process to achieve the desired accuracy depends on the system’s complexity, the particular numerical DFT implementation, the SCF algorithm used, and the initial trial electron density. Systems presenting a band gap are typically easier to converge than metals since oscillations in the electron density during the iterative convergence are largely suppressed. Such oscillations can be damped by selecting appropriate ways to update the charge density from one iteration to the next, a process that, in general, largely determines the rate at which convergence is achieved. Note that depending on the specific DFT numerical implementation, one may decide to mix the KS Hamiltonian instead of the charge density. In any case, regardless of the quantity chosen for the SCF algorithm, typically the output of several previous iterations is combined to determine the new input. The direct inversion of the iterative subspace (DIIS) method, proposed by Pulay,^19,20^ is probably the most widely used SCF solver in modern local-orbital DFT codes, where one mixes the Fock matrices. However, there exist a multitude of alternative schemes and refinements whose performance depends on the specific problem at hand.^21−29^

Regardless of the mixing method used, an initial guess for the charge density close to the final self-consistent solution will speed up convergence by typically reducing the number of iterations to perform. Such an initial density is usually defined either over a real-space or a *k*-space grid in DFT codes based on plane waves or in the form of a one-particle density matrix (DM) for local-orbital codes.^30^ There are multiple strategies to generate the initial charge density (or the DM), which all reduce to solve an associated nonself-consistent problem of some kind. As several such schemes will be explored here, a detailed description will be provided in our Methods section.

The main aim of our work is to construct ML models, namely, neural networks, to learn the ground-state one-particle DM of a DFT calculation. The models are based on structural and atomic information alone, namely, the chemical nature and positions of the atoms forming a molecule. The so-constructed DM can then be used either as a starting point for a SCF cycle or, if the accuracy is good enough, to perform a nonself-consistent evaluation of the various observables, for instance, energy and forces.

Note that ML schemes to generate the charge density in either real space^31−34^ or over an atom-center basis^35^ have already been proposed. One can then, in principle, take one of such models and try to construct the DM from the computed charge density. This strategy, however, requires projection across different incomplete basis sets, a process that inevitably introduces additional errors. These are likely to be large enough to preclude the use of the ML DM; namely, it will be unlikely to be competitive with other initial-density generation approaches. Furthermore, learning the DM directly enables straightforward evaluation of the expectation values of all one-particle operators. These include nonlocal potentials, so that the same scheme can be used with Hartree–Fock calculations. Note that a mapping between the external potential and the DM, constructed over a kernel ridge regression, has been recently proposed.^36^ This is complementary to our work since it requires smaller training sets for similar-sized molecules, but the inference is significantly more demanding. Alternatively, there is a body of literature looking at the construction of the Hamiltonian, most typically the KS one, from an equivariant description of the molecule’s structure. This is typically achieved through either an equivariant network or data augmentation. In both cases, the result is that of having rather large and deep models, which need to be trained over extensive data sets.^37−39^

This paper is organized as follows. In the next section, we discuss the main methodological aspects of the model construction and the DFT implementation used to generate the data and benchmark the results. Then, we proceed by presenting the results. In particular, we first evaluate the quality of our DM as the starting point for a SCF cycle, and then we analyze the error on the predicted energy and forces. With these results at hand, we perform nonself-consistent ab initio molecular dynamics, whose results are compared with the fully converged one. Finally, we conclude and suggest possible future directions.

## 2 Methods

As discussed in the Introduction, our task is to predict the converged DM of a DFT calculation by using a ML model, which utilizes only chemical and structural information about a molecule. In particular, we consider fully connected dense neural networks together with global structural descriptors, and we predict all the independent matrix elements of the DM. The models are defined by the neural-network architecture, the descriptor types, and the content of the training and test sets, all aspects that will be described here in detail.

**Figure 1:**
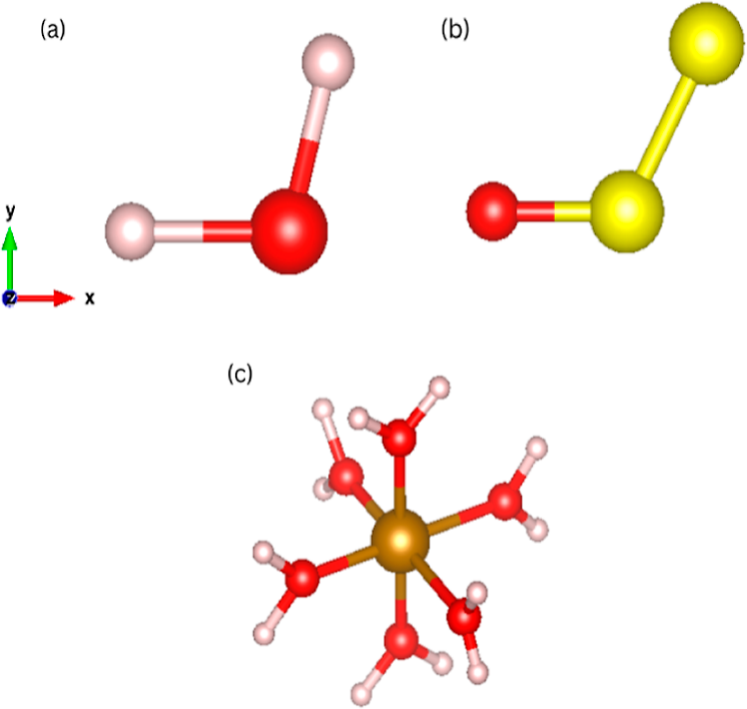
Molecules investigated in this study placed in their default positions along the Cartesian axes: (a) H_2_O, (b) S_2_O, and (c) . Color code: H = white, O = red, Fe = dark golden, S = yellow, *x*-axis = red, *y*-axis = green, and *z*-axis = blue.

All the electronic structure theory calculations performed in this work to generate the training data set and benchmark the results have been produced with the open-source Python package, PySCF.^11,40^ PySCF implements all-electron DFT and a number of quantum-chemistry methods, such as Hartree–Fock, over a Gaussian basis set. For all our calculations, we have used the cc-pVDZ basis,^41^ formed by double-ζ polarized orbitals for the valence electrons. Thus, for the atomic species relevant for our tests, we have 5 basis functions for H, 14 for O, 18 for S, and 43 for Fe. In fact, three different molecules have been considered in this work, namely, H_2_O, S_2_O, and , see Figure 1.

We start our analysis with H_2_O (*C*_2*v*_ symmetry) since this presents a simple electronic structure and typically no convergence problems. Its charge density, however, does not differ much from a superposition of atomic densities, so a more stringent test is provided by S_2_O (also *C*_2*v*_ symmetry). Importantly, both H_2_O and S_2_O can be described by only three structural features, so that the more complex  (*O*_*h*_ symmetry) is investigated last. The  molecule also gives us the opportunity to test our method for a metal bond. All electronic structure calculations are performed with the BLYP functional, which combines the generalized gradient approximation for the exchange energy of Becke^42^ with the Lee–Yang–Parr correlation energy,^43^ as implemented in the libxc library.^44^ When creating the training, validation, and test sets, the SCF cycle is converged with the DIIS scheme^19,20^ for H_2_O and S_2_O, while for , we employ a second-order solver (SOS)^28,29^ as the convergence appears more problematic. In contrast, when analyzing the convergence history of different initial DM guesses, we will consider both the DIIS and SOS SCF algorithms.

Since our ML DM will be compared with that generated by conventional initial guesses, it is worth spending some time describing them. Possibly, the simplest choice constructs the charge density as a superposition of atomic densities, while the DM is obtained from the orbitals that diagonalize the Fock matrix associated with such spin-restricted guess density. This is the “minao” PySCF default option.^45,46^ Alternatively, one can use the eigenstates of the noninteracting problem, namely, those orbitals that diagonalize a Hamiltonian comprising only the kinetic energy and the nuclear potential. This is the one-electron DM, “1e” option in PySCF, which usually represents a poor starting point for molecules.^30^ Then, there are options based on spin-restricted atomic Hartree–Fock calculations, employing different recipes for the construction of the guess orbitals used to compute the DM. In PySCF, these are called “atom”^47^ and “huckel”.^30^ Finally, one can construct the DM with the orbitals obtained from the solution of a superposition of tabulated atomic potentials.^47^ This is the “vsap” option.^30^

Here, we predict the initial guess DM with dense neural networks where the input features are the independent Cartesian coordinates of the molecules. A summary of the structure of the different neural networks, together with the training-set errors, is reported in Table 1. The use of the Cartesian coordinates together with a dense neural network effectively forces an equivariant quantity, namely, the DM, to be described by an invariant model. This issue is resolved here by manually removing the rotational and translational degrees of freedom of the molecule, a procedure that makes the entire problem invariant. Of course, such a solution is not general and a more elegant way to tackle the problem would be to use a fully equivariant representation of the molecular structure.^48^ This, however, adds a significant new layer of complexity, which we would like to avoid for our early study. Thus, we remove the translational degrees of freedom by fixing a given atom at the origin. In particular, we use oxygen, the central sulfur, and the Fe^2+^ cation, respectively, for H_2_O, S_2_O, and . Then, the rotational degrees of freedom are imposed by selecting an appropriate rotation. For the triatomic molecules, H_2_O and S_2_O, we constrain one atom on the negative *x*-axis and the second one in the *x*–*y* plane so that the molecules are described by only three coordinates. In contrast, for , we force the O atoms of the H_2_O ligands to be on the three Cartesian axes, and we consider only variation in the Fe–O bond length (the water molecules are taken as rigid). This returns us six independent coordinates (Figure 1).

**Table 1:** Summary of the Structure and Performance of the Final Neural Networks Trained for the Three Molecules^a^

molecule	*N*_in_	*D*_DM_	*N*_hi_	*N*_nu_	(*N*_w_)	MAE	δρ_max_	RMSE	*R*^2^
H_2_O	3	24 × 24	2	18, 32	662	0.0002	0.0057	0.0003	0.9999
S_2_O	3	50 × 50	2	18, 28	583	0.0002	0.0112	0.0003	0.9999
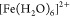	6	187 × 187	2	16, 32	640	0.0002	0.0283	0.0005	0.9993

a Here, we report the number of features defining the input, *N*_in_; the dimension of the DM, *D*_DM_; the number of the network hidden layers, *N*_hi_; the total number of neurons forming each hidden layer, *N*_nu_; and the total number of weights, *N*_w_. Then, we report the mean absolute error (MAE); the largest error on the matrix elements, δρ_max_; the root-mean square error (RMSE); and the *R*^2^ coefficient of the DMs. All errors refer to the test sets, and they are in atomic units (au).

The structure of the neural network has been optimized by varying the number of hidden layers and their size by minimizing the mean absolute error (MAE). The optimal configuration for each molecule is reported in Table 1. Note that in all cases, we employ the exponential linear unit activation function. The data sets used to construct the model are formed by 9000 configurations for training, 800 for validation, and 1000 for testing. In the case of H_2_O and S_2_O, such configurations are extracted from ab initio Born–Oppenheimer molecular dynamics trajectories at 150 K, also performed with PySCF. In particular, we run for 117 and 130 ps, respectively, for H_2_O and S_2_O, by using the Nosé–Hover thermostat through the pyLammps API as implemented in the LAMMPS package.^49^ In contrast, for the case of , we consider random rigid displacement of the H_2_O molecules, such that the Fe–O bond length is varied within 10% from its equilibrium value (2.0525 Å). These return us training-set MAEs of the order of 10^–3^ atomic units (au). Note that typically, the largest DM elements are found along the diagonal, and they can reach values close to unity, while a significant fraction of the off-diagonal matrix elements remain small. For example, for the H_2_O molecule, we find 8.15% of the matrix elements, ρ, having values 0.1 < |ρ| < 1, 43.75% in the range 10^–3^ < |ρ| < 0.1 and –48.1% being |ρ| < 10^–3^. The parity plots associated with our neural networks, together with the typical matrix element distributions, can be found in the Appendix for all three molecules. Note also that our numerical construction of the DM does not guarantee idempotency to be satisfied, and in fact, we find that this is numerically violated. Since idempotency is a nonlinear condition, it is difficult to implement it as a constraint in the neural networks. Such a drawback is compensated here by the numerical accuracy achieved, as we will demonstrate in the following.

Finally, we perform tests on how the computed DM can drive molecular dynamics; namely, we perform ab initio molecular dynamics using our ML DM and not the one resulting from a SCF cycle. In this case, special care must be taken since the neural networks return DM only for molecules having the specific spatial orientations described before. For this reason, we operate the following workflow. A molecule at an arbitrary position is translated back to its origin and rotated so as to have the orientation required by the neural networks. Then, the DM is evaluated with the network and used as a starting guess for a static DFT calculation (nonself-consistent). Energy and forces are thus evaluated using PySCF. The molecule is then rotated/translated back to its original position, and the same rotation is applied to the forces. Such a force field is input into the molecular dynamics package, which updates the atomic coordinates. Then, the process is repeated. The molecular dynamics steps are implemented in the LAMMPS package.^49^ Although more cumbersome than standard molecular dynamics, the strategy adopted here allows us to use our simple structural descriptors for a problem: the construction of the DM, which is intrinsically translational invariant and rotational covariant. Translational invariance can be achieved by using local structural descriptors, while rotations can be accounted for with a covariant model. Here, we have preferred to keep our model as simple as possible to concentrate fully on demonstrating how a DM can be constructed with ML.

## 3 Results and Discussion

In order to validate our entire approach, we perform three different tests. First, we evaluate the efficacy of the ML DM as a starting point for an SCF cycle. Then, we quantify the accuracy of the DM in determining the energy and forces. Finally, we compare the molecular dynamics trajectories driven by the forces associated with the ML DM with those of fully self-consistent ab initio molecular dynamics.

### 3.1 ML DM as an Initial Guess

**Figure 2:**
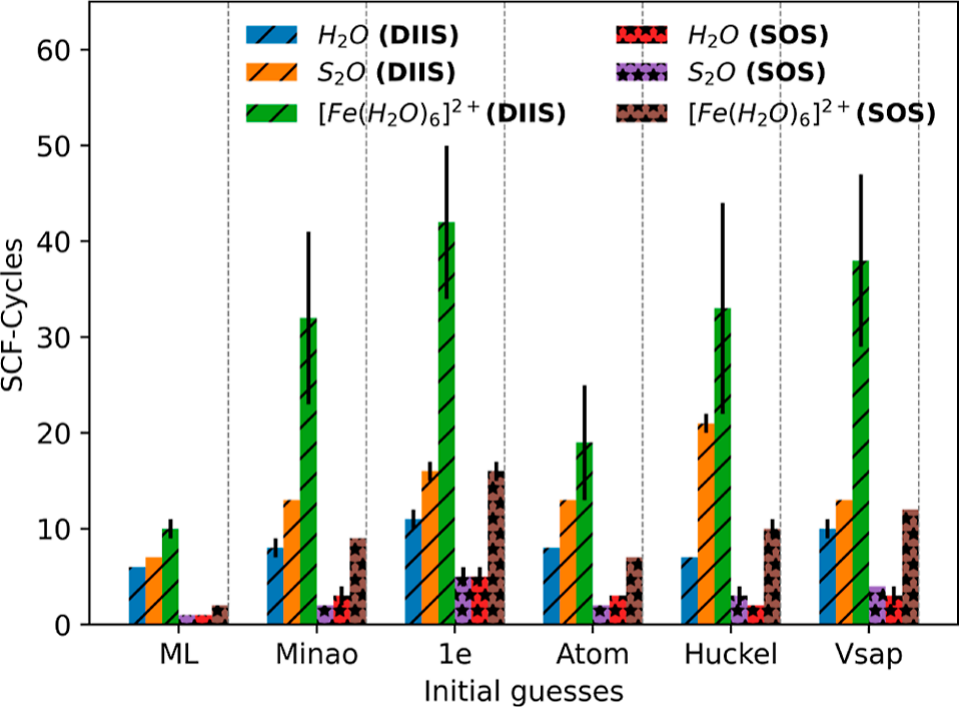
Total average number of SCF steps taken to achieve convergence for different starting DMs and mixing schemes. Here, the convergence criterion is on the total energy between two consecutive SCF steps that should be lower than 10^–9^ Ha. The black bars around the mean indicate the variance. Variances lower than one iteration are not displayed.

The first test consists of evaluating how accurate the DM generated by the neural networks is as a starting point for the DFT SCF cycle. The test is performed over 1000 new configurations for each molecule, and in Figure 2, we report the average number of SCF iterations performed to achieve convergence and their associated variance. In this case, convergence is defined by having an energy difference between subsequent iterations lower than 10^–9^ Ha, a value that sets a rather tight convergence criterion. For this test, we perform two sets of calculations, where the SCF cycles are driven, respectively, by the DIIS or the SOS mixing scheme, with convergence parameters set by the PySCF default.

In general, we find the SOS DM-update strategy to be significantly more performing than the simpler DIIS, with the total number of iterations reducing by approximately a factor of three regardless of the molecule or the initial DM. Note that this advantage is partially compensated by the SOS scheme being numerically heavier than DIIS; namely, a single iteration takes longer. We have also found that sometimes for  and the DIIS solver, 50 iterations are not enough to achieve convergence. This is somehow expected considering the electronic structure of the  cation. In fact,  is a spin-crossover molecule presenting a temperature-induced low-spin to high-spin transition, driven by a distortion of the octahedral coordination shell of the Fe^2+^ ion. This is only partially described by DFT,^50^ and a multideterminant theory appears more appropriate.^51^ For this reason, it is not surprising that for some highly distorted configurations, our nonspin-polarized DFT calculations struggle to converge. Note that this is not so crucial here since we are not seeking to compute the exact ground state of  but simply to present a test example for “difficult” convergence. In any case, when convergence is not achieved, the SCF cycle is stopped after 50 iterations.

Despite these differences, the convergence-speed trends with respect to the initial DM are rather similar across the two mixing schemes, so in our discussion, we refer first to data obtained with the SOS algorithm. As expected, the H_2_O and S_2_O molecules converge significantly faster than , as their simple covalent bonding structure would suggest. Also, as expected, the simple “1e” default provides the worst starting DM, and convergence is achieved in five iterations for H_2_O and S_2_O and about 16 for . The other conventionally constructed starting DMs appear to perform rather similarly to each other with the two covalently bonded molecules converging in about 3–4 SCF steps and  in about 10. Most importantly, our DM significantly outperforms any other methods, with a single SCF iteration being necessary for H_2_O and S_2_O, while  requires only two. This gives us a speedup in the computation of the SCF cycle comprised between a factor of 3 and a factor of 5. Note that the speedup is less pronounced when the DIIS mixing scheme is used, in particular, for the covalently bonded molecules, where the advantage over the other schemes is only fractional. This difference seems to boil down to the inefficiency of the mixing scheme, which brings the iteration count to 6–7 even when the calculation is initiated with the ML DM. While it is not straightforward to establish why the SOS algorithm offers a better convergence speedup to ML-generated initial DMs’, we note here that, in general, DIIS algorithms may not honor well the initial guess, meaning that the optimization procedure may lead the electron density anywhere in the variational space. It is then expected that such a drawback penalizes more DMs close to the fully converged one than more inaccurate ones.

### 3.2 Convergence Analysis

**Figure 3:**
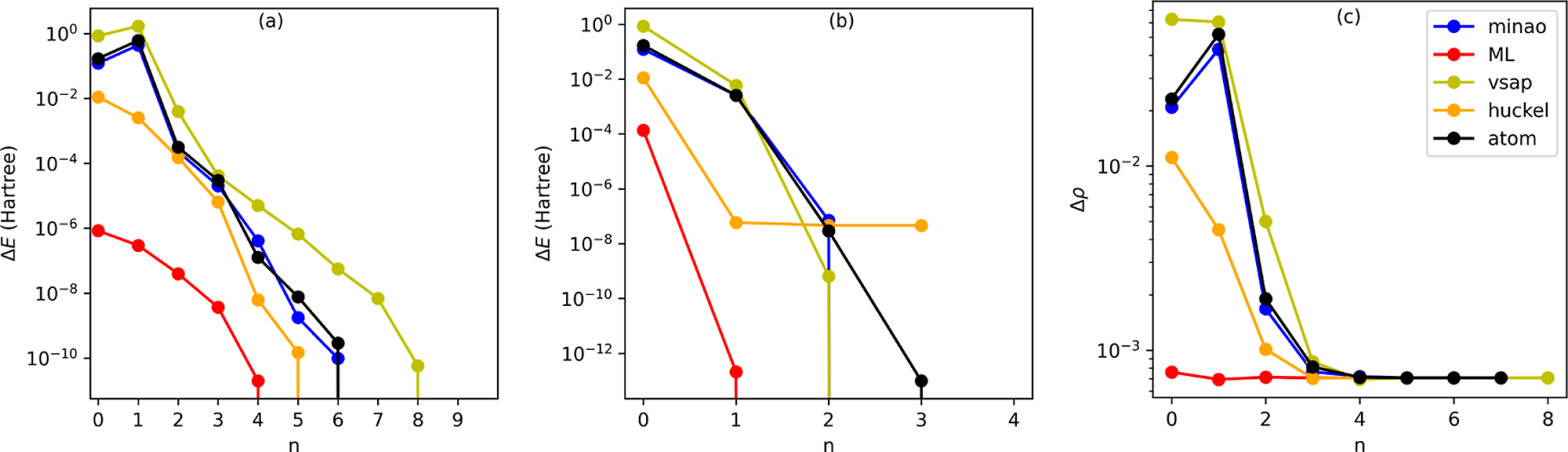
Analysis of the SCF cycle for H_2_O. In panels (a,b), we show the total energy (measured with respect to the ground-state energy) as a function of the iteration number, *n*, for convergence driven by the DIIS and SOS mixing schemes, respectively. In panel (c), we present the norm of the difference between the ground-state converged DM, ρ^GS^, and that computed at the *n*th iteration, ρ^*n*^. In this case, we follow the DIIS-driven SCF cycle. For ease of visualization, in all plots, the *y* axis is on a logarithmic scale.

We now look in more detail at how convergence is achieved for different starting DMs and the two different mixing schemes. We begin by considering H_2_O and then move to . The results for S_2_O are somehow between these two cases and are presented in the Supporting Information . In Figure 3, we show the total energy (with respect to the ground-state energy) as a function of the iteration number, *n*, for H_2_O computed with the DIIS [panel (a)] and SOS [panel (b)] mixing schemes. Furthermore, in panel (c), we present the norm of the difference between the ground-state (converged) DM, ρ^GS^, and that at the *n*th iteration, ρ^*n*^, also along the DIIS-driven SFC cycle. This last quantity is computed as , with ρ_*ij*_ being the *i*, *j* DM element. Although the details of each SCF cycle may differ depending on the molecule geometry, the figure shows a typical case.

In general, all initial DMs are somewhat different from the final ground-state one, with the largest variations found, as expected, for the “1e” initialization (the total energy difference at *n* = 0 is in excess of 8 Ha, and it is not displayed here). The convergence is then monotonic when the SOS solver is used, while it may present oscillations for DIIS. This explains the largest number of iterations typically taken by DIIS. Strikingly, the ML-generated DM appears extremely close to the final ground-state one, so that the convergence is monotonic in all cases. In fact, the *n* = 0 computed total energies for H_2_O are on average within 10^–4^ Ha from their ground-state value, and the percentage variation of the DM at the first iteration is only 0.196%. This suggests that, by and large, the ML DM already provides an excellent estimation of the ground-state DM. As a comparison, the second-best initial DM appears to be that generated with a restricted Hartree–Fock calculation (“huckel” option), with an initial total energy error of about 10^–2^ Ha. All the other DM-generating schemes have an initial error larger than 0.1 Ha.

**Figure 4:**
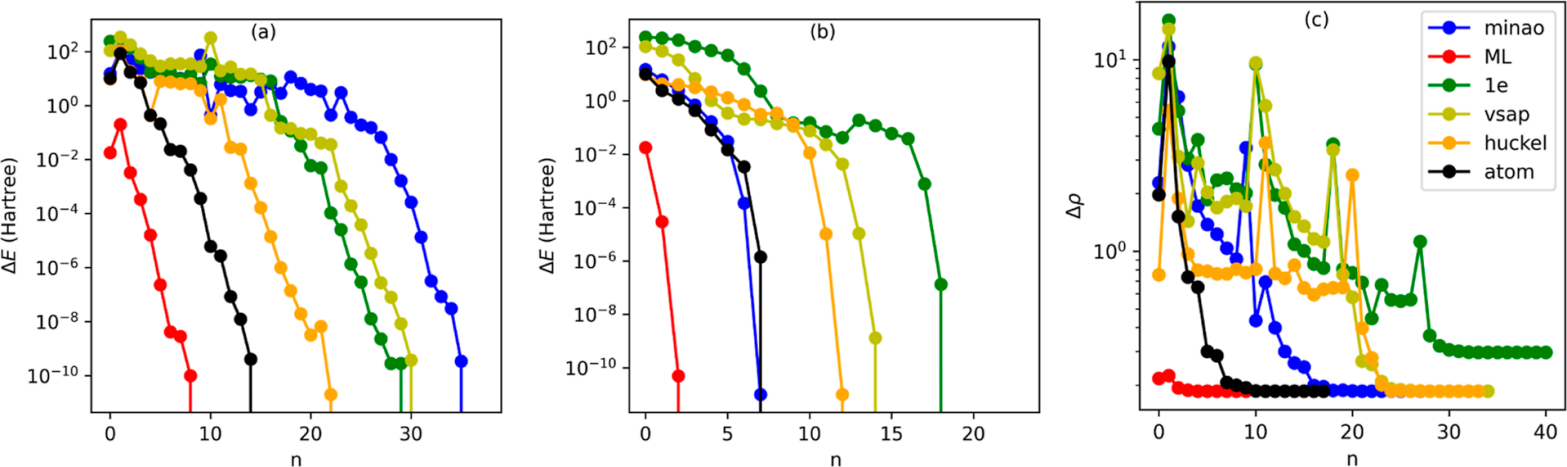
Analysis of the SCF cycle for . In panels (a,b), we show the total energy (measured with respect to the ground-state energy) as a function of the iteration number, *n*, for convergence driven by the DIIS and SOS mixing schemes, respectively. In panel (c), we present the norm of the difference between the ground-state converged DM, ρ^GS^, and that computed at the *n*th iteration, ρ^*n*^. In this case, we follow the DIIS-driven SCF cycle. For ease of visualization, in all plots, the *y* axis is on a logarithmic scale.

The path to convergence becomes significantly more oscillatory when one looks at the DIIS SCF cycle for  (see Figure 4). This time, the energy and DM fluctuations are significantly more pronounced, with the appearance of “spikes” in correspondence to some self-consistent steps when using the DIIS solver. These originate from fluctuations in the atomic orbital occupation across different SCF iterations. Such large fluctuations are suppressed by the SOS mixing scheme, which reinstates a monotonic approach to the ground-state solution. Most importantly, also for , the ML-constructed DM provides a much more accurate starting point. In fact, it is sufficiently accurate that the oscillations are suppressed, regardless of the mixing scheme. Furthermore, already at the first iteration, energy and DM are extremely accurate. This time, the average energy is about 3 × 10^–5^ Ha away from the converged one (this corresponds to an error of 1.7 × 10^–6^%), while the percentage variation of the DM at the first iteration with respect to the ground state is 0.77%.

### 3.3 Nonself-Consistent Forces

The previous section has shown that the ML DM requires an extremely limited number of SCF iterations to achieve the desired convergence and that even without any iteration, it can already provide an accurate estimate of the DFT total energy. Here, we explore further this second aspect and investigate the accuracy of our ML DM in predicting a second observable, namely, the atomic forces. For this section, we consider the S_2_O molecule in particular, but the results for H_2_O and  are qualitatively rather similar and are presented in the Supporting Information.

**Figure 5:**
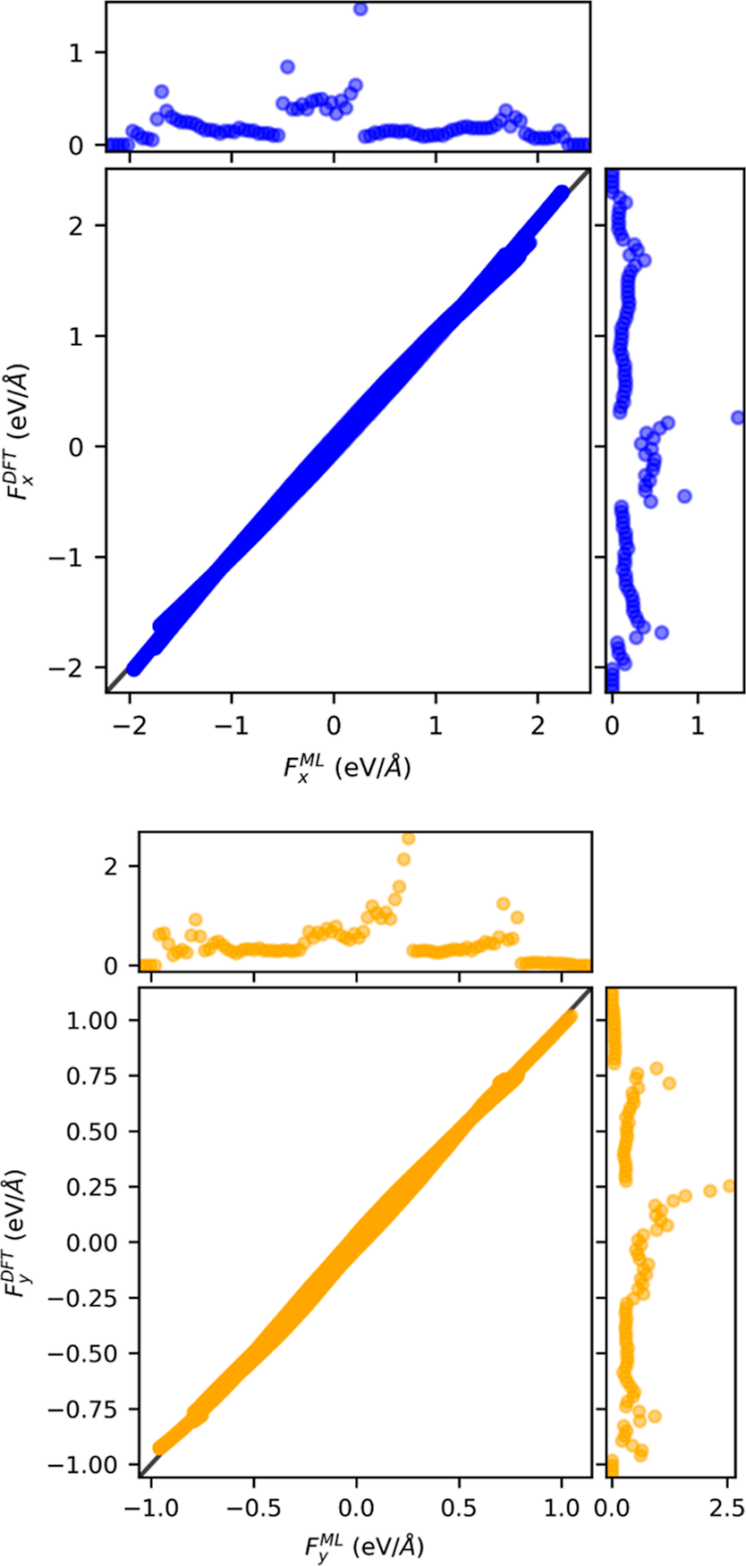
Parity plot for the α = *x*, *y* component of the atomic forces computed by using the ML DM, *F*_α_^ML^, with one SCF cycle, against the fully converged DFT ones, *F*_α_^DFT^. Data are here presented for a set of 1000 S_2_O molecules extracted from the same molecular dynamics trajectory used to generate the training set. The upper panel is for the forces *x* component, while the lower panel is for the *y* component. The histograms on the side describe the frequency of the forces in the test set.

In Figure 5, we present the parity plot diagram for the *x* [upper panel] and *y* [lower panel] components of the atomic forces acting on the atom lying in the *x*–*y* plane. These are computed for a set of 1000 distorted molecules obtained from the molecular dynamics trajectory used to generate the training set but never used in the construction of the neural network. Since the molecules are, by construction, always aligned in the *x*–*y* plane, there are no forces along *z*. The parity plot compares the fully converged DFT forces (*y* axis) with those predicted from the ML DM without operating any SCF iteration (*x* axis). Points on the parity line are predicted exactly. The graphs also show histograms of the distributions of the atomic forces. Note that along our molecular dynamics trajectory, the forces can be as large as 2 eV/Å, but typically, they are concentrated within ±0.25 eV/Å.

Clearly, there is extremely good mapping between the ML-DM-predicted forces and the exact ones, with the vast majority of the points staying on the parity line. This is reflected in the almost identical force distributions. Quite interestingly, there is no biased distribution of errors across the range of force magnitude explored, in contrast to what is usually found for ML force fields, where the largest error is encountered for small forces. The MAE is calculated at 126 and 62 meV/Å, respectively, for the *x* and *y* components. Such an error can be further reduced by noticing that the ML-generated DM does not necessarily describe an integer number of electrons. This feature can be corrected by rescaling the ML-generated DM of a factor *N*_e_/*N*_e_^ML^, where *N*_e_ is the total number of electrons and *N*_e_^ML^ that computed using the as-generated ML DM, *N*_e_^ML^ = Tr[ρ^ML^·*S*], with *S* being the overlap matrix. For S_2_O, we find that typically *N*_e_^ML^ is less than 0.1% different from *N*_e_, but the correction is enough to bring down the MAE to 62 and 22 meV/Å, respectively, for the *x* and *y* components (the parity plots of Figure 5 have been obtained with the forces computed after such DM rescaling). This error is significantly lower than what is typically found for state-of-the-art force fields.^52−54^ Although a thorough comparison is not simple since the analysis needs to be carried out with the same molecules, the same training set size, etc., this result clearly demonstrates that predicting the DM to be used in nonself-consistent DFT can be a valid alternative to the construction of a force field. Namely, the forces obtained from the ML-predicted DM can be used as a driver for molecular dynamics. This aspect is explored last in the next section.

### 3.4 Nonself-Consistent Molecular Dynamics

As a final test, we now perform molecular dynamics simulations by using the ML-predicted DM. In particular, we use the forces obtained after rescaling the DM by *N*_e_/*N*_e_^ML^ and after a single SCF step. Such a step is needed since the rescaled DM seems to have a total energy marginally lower than that of the ground state. The molecular dynamics simulations are then performed at 150 K for S_2_O and H_2_O for a total of 0.14 and 0.12 ns, respectively. In both cases, we use the rotation procedure described in the Methods section to avoid the need for an equivariant model. The trajectories obtained are then compared with the fully converged ab initio ones and with those computed by performing only a single DIIS self-consistent step, starting from the PySCF default “minao” charge density.

**Figure 6:**
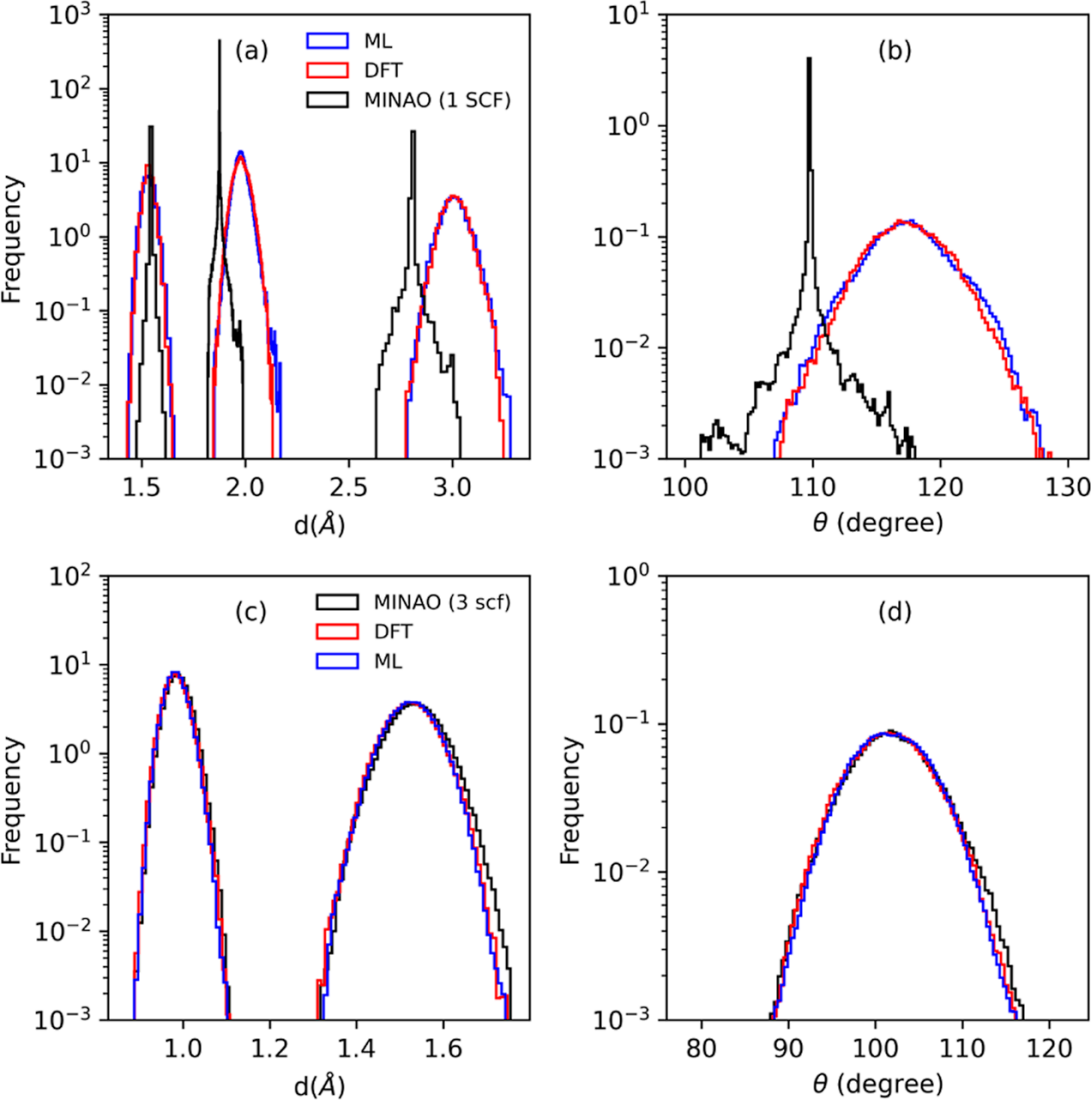
Histograms of the bond lengths, *d*, and bond angles, θ, along the molecular dynamics trajectories for S_2_O [panels (a) and (b)] and H_2_O [panels (c) and (d)]. There are two distinct bond lengths for H_2_O, namely, O–H and H–H, while there are three for S_2_O, namely, two S–O and one S–S.

The different molecular dynamics trajectories are monitored and compared by looking at the bond length and bond angle thermal distributions, which are presented in Figure 6 for the two molecules. In the case of H_2_O [panels (c) and (d)], there is no significant difference between the various methods, with rather similar distributions. This has to be expected considering the speed of convergence of the SCF cycle in this case. Thus, we find an average O–H bond length of about 0.9837 Å and an average bond angle of 101.83°, values fully consistent with the static DFT BLYP results, 0.9751 Å and 104.14°, and with the experimental values of 0.9578 Å and 104.47°.^55^

**Figure 7:**
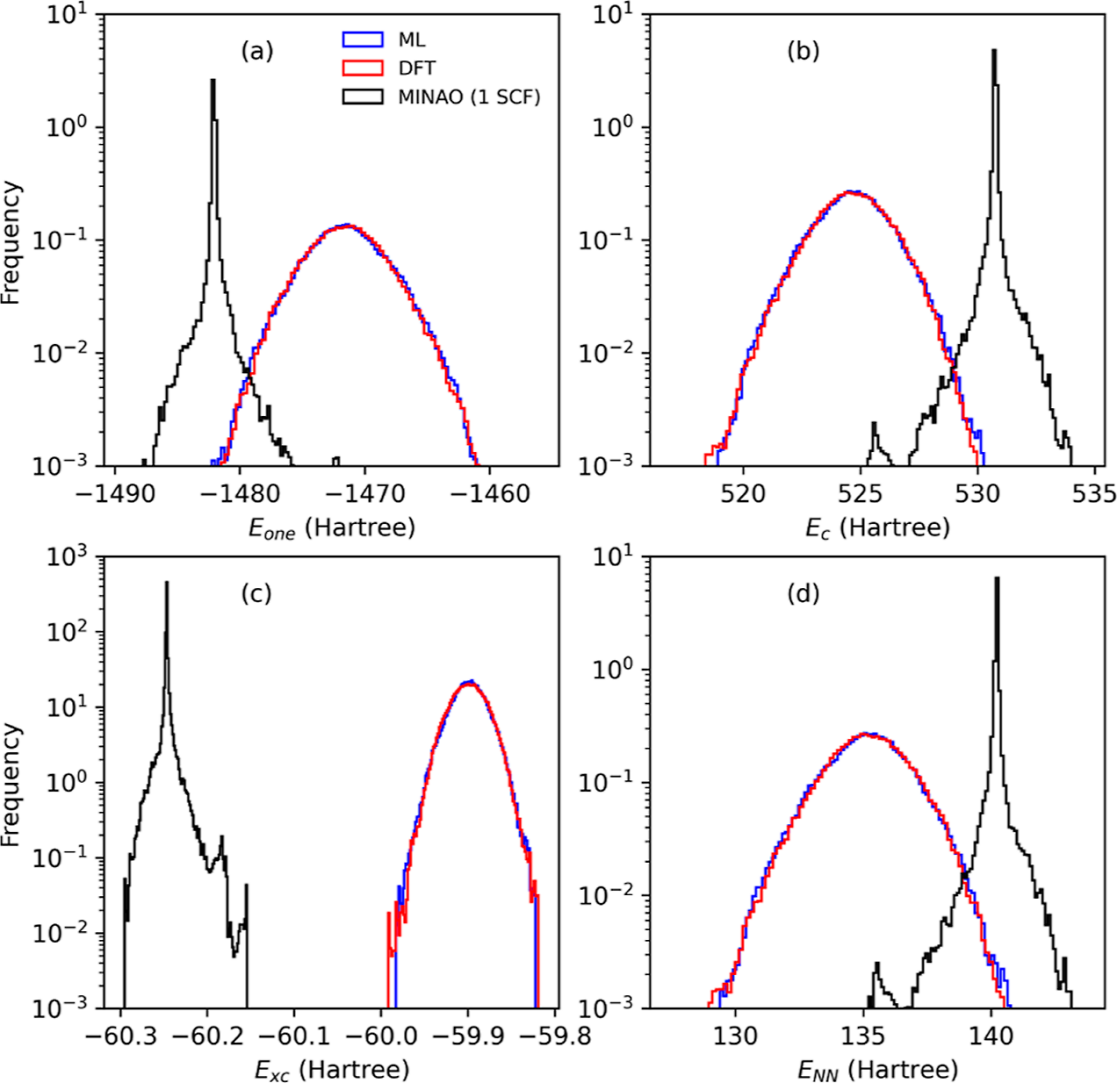
Histogram of the various energy components along the different molecular dynamics trajectories for the S_2_O molecule. Here, we separate the total energy into one-electron, *H*_one_, Coulomb (Hartree), *H*_C_, exchange–correlation, *H*_XC_, and nucleus–nucleus, *H*_NN_, components.

The S_2_O case is, instead, quite different. From panels (a) and (b) of Figure 6, one can appreciate that the ML DM provides an excellent estimate of the fully DFT-converged one, so that the thermal distributions of bond lengths and angles are rather similar to those obtained with fully ab initio molecular dynamics. In this case, there are three bond lengths corresponding to the S–O bond, the S–S one, and the second S–O distance between the two most peripheral atoms. The centers of the distributions are close to the reported experimental values of 1.4650 Å (S–O), 1.8834 Å (S–S), and 3.2505 Å (S–S),^55^ and so is the bond angle, 117.876°, with the remaining differences being attributed to the choice of DFT exchange and correlation energy. This is not the case when the molecular dynamics is performed with a single SCF step starting from the PySCF “minao” initialization. In fact, the low accuracy in the determination of the forces results in an average structure presenting a significantly compressed S–S bond and a drastic reduction in the bond angle. Finally, in Figure 7, we present the decomposition of the total energy over the one-electron, *H*_one_, Coulomb (Hartree), *H*_C_, exchange–correlation, *H*_XC_, and nucleus–nucleus, *H*_NN_, components. As expected, since the average structures are erroneously predicted, the minimally initialized molecular dynamics provides distributions pretty far from those obtained with self-consistent DFT. This contrasts with the results obtained from our ML DM, which describes not only the structure well but also all energy components.

## 4 Conclusions

We have shown that neural networks can be trained to predict the DM required by electronic structure theories constructed over atomic-orbital basis sets. These ML DMs are of sufficient quality to be used as initial guesses in KS DFT, demonstrating a reduction in the number of self-consistent steps needed to achieve convergence over all other common initial choices. Equally important, such DMs return rather accurate energy and forces even without self-consistency, a fact that enables one to run inexpensive molecular dynamics simulations whose computational cost is similar to that needed by ML force fields. We have shown here results for three molecules, H_2_O, S_2_O, and , and DFT, but the method is agnostic to the system to describe and the choice of electronic structure theory, as long as this is based on the DM. For instance, it can be employed together with wave function-based quantum-chemistry methods such as Hartree–Fock. It is also important to note that although here the molecule structure is represented by simple Cartesian coordinates, our proposed method can be implemented with more sophisticated structural descriptors. These can be constructed equivariantly^56^ and can be descriptive enough to avoid the need for using deep-learning ML models.^54^

The drawback of our scheme is that the number of matrix elements to predict scales quadratically with the number of basis functions used in the calculation so that it becomes increasingly expensive as the system size grows. In practice, many of the matrix elements remain rather small, and they can be safely neglected when evaluating the DM for accurate, nonself-consistent electronic structure or molecular dynamics. Furthermore, efficiency can be achieved by constructing the ML DM over a small basis set and then using it for calculations employing larger ones.^30^

It is important to note, however, that whether or not a ML strategy for the construction of the DM is favorable against other solutions ultimately depends on many considerations. These are characteristics of the system to be investigated and the workflow adopted. In particular, one may consider three determining factors: (1) the size of the training set needed, namely, how many DFT calculations one needs to perform for constructing the model; (2) the size of the optimal neural network as the dimension of the DM grows (heavier networks may be required as the complexity increases); and (3) the workflow in which the method is deployed, namely, how many calculations one has to perform once the ML model has been constructed. All these factors together determine the “computational economy” of any ML approach, and a careful assessment must be carried out before a specific computational strategy is selected.

In any case, our scheme will become progressively more convenient as the scaling of the overarching electronic structure method with the system size becomes more prohibitive. In this case, the quadratic scaling of the DM construction is overshadowed by the computational cost to run long, self-consistent cycles, and significant savings in computational overhead can be achieved. This can be the case, for instance, of nonlocal exchange–correlation functionals.

## Figures and Tables

**Figure 8:**
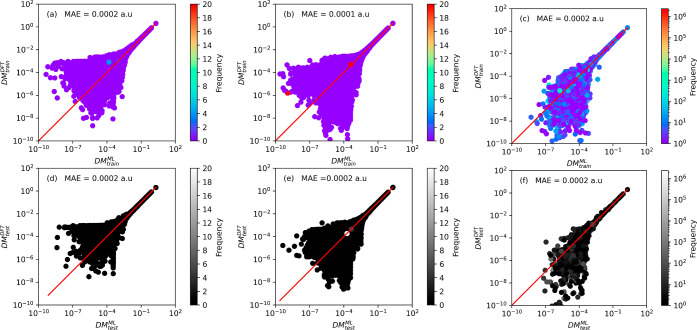
Parity plots for H_2_O [panels (a,d)], S_2_O [panels (b,e)], and  [panels (c,f)]. The upper panels are for the training set and the lower ones for the test one. Each graph also reports the MAE achieved. Note that all the parity plots are in the logarithmic scale (we plot |ρ_*ij*_|) and that deviations are only found for the smaller matrix elements. The color code describes the density of given DM-element values.

## A Neural-Network Parity Plots

In Figure 8, we present the parity plots (in logarithmic scale) for the elements of the DM of H_2_O [panels (a) and (d)], S_2_O [panels (b) and (e)], and  [panels (c) and (f)]. The upper panels are for the training set and the lower ones for the test set, and the value of the MAE is reported in all cases. As we can see, there is excellent agreement between the ML-computed DM and the fully converged DFT one, with most of the points lying on the parity line. This is true for matrix elements larger than ∼10^–2^, namely, for those that have a dominant behavior on all the observables of relevance (e.g., total energy and forces). Larger relative errors are found for smaller elements, for which the DFT data are already noisy and the ML model can hardly train. This distribution of errors reflects the rather low MAE reported for all cases (see also Table 1). The only exception is for a few points in the training set of , for which some more pronounced deviations are reported. These, however, correspond to situations where the DFT calculations struggle to converge (see main test) and the DM is the one obtained at the maximum number of self-consistent iterations allowed (hence, not the converged one). These training points are thus discarded. The test set, instead, contains only examples where full convergence is obtained.

## Supporting Information Available

Convergence analysis for S_2_O and nonself-consistent energy and forces for H_2_O and  (PDF)

## References

[ref1] Hohenberg P.; Kohn W. Inhomogeneous electron gas. Phys. Rev. 1964, 136, B864–B871. 10.1103/PhysRev.136.B864.

[ref2] Kohn W.; Sham L. J. Self-consistent equations including exchange and correlation effects. Phys. Rev. 1965, 140, A1133–1138. 10.1103/PhysRev.140.A1133.

[ref3] Parr R. G.; Yang W. Density-Functional Theory of Atoms and Molecules; Oxford University Press: New York, 1995.

[ref4] Perdew J. P.; Schmidt K. Jacob’s ladder of density functional approximations for the exchange-correlation energy. AIP Conf. Proc. 2001, 577, 1–20. 10.1063/1.1390175.

[ref5] Kresse G.; Hafner J. Ab initio molecular dynamics for liquid metals. Phys. Rev. B 1993, 47, R558–R561. 10.1103/physrevb.47.558. 10004490

[ref6] Giannozzi P.; Andreussi O.; Brumme T.; Bunau O.; Buongiorno Nardelli M.; Calandra M.; Car R.; Cavazzoni C.; Ceresoli D.; Cococcioni M.; et al. Advanced capabilities for materials modelling with Quantum ESPRESSO. J. Phys.: Condens. Matter 2017, 29, 465901 10.1088/1361-648X/aa8f79. 29064822

[ref7] Blaha P.; Schwarz K.; Tran F.; Laskowski R.; Madsen G. K. H.; Marks L. D. WIEN2k: An APW+lo program for calculating the properties of solids. J. Chem. Phys. 2020, 152, 074101 10.1063/1.5143061. 32087668

[ref8] Romero A. H.; Allan D. C.; Amadon B.; Antonius G.; Applencourt T.; Baguet L.; Bieder J.; Bottin F.; Bouchet J.; Bousquet E.; et al. ABINIT: Overview and focus on selected capabilities. J. Chem. Phys. 2020, 152, 124102 10.1063/1.5144261. 32241118

[ref9] Blum V.; Gehrke R.; Hanke F.; Havu P.; Havu V.; Ren X.; Reuter K.; Scheffler M. Ab initio molecular simulations with numeric atom-centered orbitals. Comput. Phys. Commun. 2009, 180, 2175–2196. 10.1016/j.cpc.2009.06.022.

[ref10] García A.; et al. Siesta: Recent developments and applications. J. Chem. Phys. 2020, 152, 204108 10.1063/5.0005077. 32486661

[ref11] Sun Q.; Zhang X.; Banerjee S.; Bao P.; Barbry M.; Blunt N. S.; Bogdanov N. A.; Booth G. H.; Chen J.; Cui Z. H.; et al. Recent developments in the PySCF program package. J. Chem. Phys. 2020, 153, 024109 10.1063/5.0006074. 32668948

[ref12] Lejaeghere K.; Bihlmayer G.; Björkman T.; Blaha P.; Blügel S.; Blum V.; Caliste D.; Castelli I. E.; Clark S. J.; Dal Corso A.; et al. Reproducibility in density functional theory calculations of solids. Science 2016, 351, aad3000 10.1126/science.aad3000. 27013736

[ref13] Lehtola S.; Marques M. A. L. Reproducibility of density functional approximations: How new functionals should be reported. Chem. Phys. 2023, 159, 114116 10.1063/5.0167763. 37725491

[ref14] Wang Y. A.; Carter E. A. Theoretical Methods in Condensed Phase Chemistry. Progress in Theoretical Chemistry and Physics, vol 5. In Orbital-Free Kinetic-Energy Density Functional Theory; Schwartz S. D., Ed.; Springer: Dordrecht, 2002.

[ref15] Chen H.; Zhou A. Orbital-free density functional theory for molecular structure calculations. Numer. Math. Theor. Meth. Appl. 2008, 1, 1–28.

[ref16] Wesolowski T. A.; Wang Y. A. Recent Progress in Orbital-Free Density Functional Theory; World Scientific (Singapore), 2013. 10.1142/8633.

[ref17] Li L.; Snyder J. C.; Pelaschier I. M.; Huang J.; Niranjan U.-N.; Duncan P.; Rupp M.; Müller K.; Burke K. Understanding machine-learned density functionals. Int. J. Quantum Chem. 2016, 116, 819–833. 10.1002/qua.25040.

[ref18] Szabo A.; Ostlund N. S. Modern Quantum Chemistry: Introduction to Advanced Electronic Structure Theory; Dover Publications: New York, 1996.

[ref19] Pulay P. Convergence acceleration of iterative sequences - the case of SCF iteration. Chem. Phys. Lett. 1980, 73, 393–398. 10.1016/0009-2614(80)80396-4.

[ref20] Pulay P. Improved SCF convergence acceleration. J. Comput. Chem. 1982, 3, 556–560. 10.1002/jcc.540030413.

[ref21] Kudin K. N.; Scuseria G. E.; Cancès E. A black-box self-consistent field convergence algorithm: One step closer. J. Chem. Phys. 2002, 116, 8255–8261. 10.1063/1.1470195.

[ref22] Kudin K. N.; Scuseria G. E. Converging self-consistent field equations in quantum chemistry - recent achievements and remaining challenges. ESAIM: M2AN 2007, 41, 281–296. 10.1051/m2an:2007022.

[ref23] Cancés E.; Le Bris C. Can we outperform the DIIS approach for electronic structure calculations?. Int. J. Quantum Chem. 2000, 79, 82–90. 10.1002/1097-461x(2000)79:2<82::aid-qua3>3.0.co;2-i.

[ref24] Saunders V. R.; Hillier I. H. A “Level–Shifting” method for converging closed shell Hartree–Fock wave functions. Int. J. Quantum Chem. 1973, 7, 699–705. 10.1002/qua.560070407.

[ref25] Bacskay G. B. A quadratically convergent Hartree-Fock (QC-SCF) method - application to closed shell systems. Chem. Phys. 1981, 61, 385–404. 10.1016/0301-0104(81)85156-7.

[ref26] Bhattacharyya S. P. Accelerated convergence in SCF calculations and the level shifting technique. Chem. Phys. Lett. 1978, 56, 395–398. 10.1016/0009-2614(78)80268-1.

[ref27] Hu X.; Yang W. Accelerating self-consistent field convergence with the augmented Roothaan-Hall energy function. J. Chem. Phys. 2010, 132, 054109 10.1063/1.3304922. 20136307 PMC2830258

[ref28] Sun Q. Co-iterative augmented Hessian method for orbital optimization. arXiv 2016, arXiv:1610.08423 10.48550/arXiv.1610.08423.

[ref29] Sun Q.; Yang J.; Chan G. K.-L. A general second order complete active space self-consistent-field solver for large-scale systems. Chem. Phys. Lett. 2017, 683, 291–299. 10.1016/j.cplett.2017.03.004.

[ref30] Lehtola S. Assessment of initial guesses for self-consistent field calculations, Superposition of atomic potentials: simple yet efficient. J. Chem. Theory Comput.h 2019, 15, 1593–1604. 10.1021/acs.jctc.8b01089. PMC6727215 30653322

[ref31] Brockherde F.; Vogt L.; Li L.; Tuckerman M. E.; Burke K.; Müller K. R. Bypassing the Kohn-Sham equations with machine learning. Nat. Commun. 2017, 8, 872 10.1038/s41467-017-00839-3. 29021555 PMC5636838

[ref32] Chandrasekaran A.; Kamal D.; Batra R.; Kim C.; Chen L.; Ramprasad R. Solving the electronic structure problem with machine learning. npj Comput. Mater. 2019, 5, 22 10.1038/s41524-019-0162-7.

[ref33] Ellis J. A.; Fiedler L.; Popoola G. A.; Modine N. A.; Stephens J. A.; Thompson A. P.; Cangi A.; Rajamanickam S. Accelerating finite-temperature Kohn-Sham density functional theory with deep neural networks. Phys. Rev. B 2021, 104, 035120 10.1103/PhysRevB.104.035120.

[ref34] Focassio B.; Domina M.; Patil U.; Fazzio A.; Sanvito S. Linear Jacobi-Legendre expansion of the charge density for machine learning-accelerated electronic structure calculations. npj Comp. Mater. 2023, 9, 87 10.1038/s41524-023-01053-0.

[ref35] Grisafi A.; Fabrizio A.; Meyer B.; Wilkins D. M.; Corminboeuf C.; Ceriotti M. Transferable Machine-Learning Model of the Electron Density. ACS Cent. Sci. 2019, 5, 57–64. 10.1021/acscentsci.8b00551. 30693325 PMC6346381

[ref36] Shao X.; Paetow L.; Tuckerman M. E.; Pavanello M. Machine Learning Electronic Structure Methods Based On The One-Electron Reduced Density Matrix. Nature Commun. 2023, 14, 6281 10.1038/s41467-023-41953-9. 37805614 PMC10560258

[ref37] Schütt K. T.; Gastegger M.; Tkatchenko A.; Müller K. R.; Maurer R. J. Unifying machine learning and quantum chemistry with a deep neural network for molecular wavefunctions. Nat. Commun. 2019, 10, 5024 10.1038/s41467-019-12875-2. 31729373 PMC6858523

[ref38] Unke O., Bogojeski M., Gastegger M., Geiger M., Smidt T., Müller K.-R. SE(3)-equivariant prediction of molecular wavefunctions and electronic densities. In Advances in Neural Information Processing Systems; The MIT Press, 2021; Vol 34

[ref39] Zhang L.; Onat B.; Dusson G.; McSloy A.; Anand G.; Maurer R. J.; Ortner C.; Kermode J. R. Equivariant analytical mapping of first principles Hamiltonians to accurate and transferable materials models. npj Comp. Mater. 2022, 8, 158 10.1038/s41524-022-00843-2.

[ref40] Sun Q.; Berkelbach T. C.; Blunt N. S.; Booth G. H.; Guo S.; Li Z.; Liu J.; McClain J. D.; Sayfutyarova E. R.; Sharma S.; Wouters S.; Chan G. K.-L. PySCF: the Python-based simulations of chemistry framework. Wiley Interdiscip. Rev.: Comput. Mol. Sci. 2018, 8, e1340 10.1002/wcms.1340.

[ref41] Dunning T. H. Jr Gaussian basis sets for use in correlated molecular calculations. I. The atoms boron through neon and hydrogen. J. Chem. Phys. 1989, 90, 1007–1023. 10.1063/1.456153.

[ref42] Becke A. D. Density-functional exchange-energy approximation with correct asymptotic behavior. Phys. Rev. A 1988, 38, 3098–3100. 10.1103/PhysRevA.38.3098. 9900728

[ref43] Lee C.; Yang W.; Parr R. G. Development of the Colle-Salvetti correlation-energy formula into a functional of the electron density. Phys. Rev. B 1988, 37, 785–789. 10.1103/PhysRevB.37.785. 9944570

[ref44] Lehtola S.; Steigemann C.; Oliveira M. J. T.; Marques M. A. L. Recent developments in libxc - A comprehensive library of functionals for density functional theory. Software X 2018, 7, 1–5. 10.1016/j.softx.2017.11.002.

[ref45] Almlöf J.; Faegri K.; Korsell K. Principles for a direct SCF approach to LICAO–MOab-initio calculations. J. Comput. Chem. 1982, 3, 385–399. 10.1002/jcc.540030314.

[ref46] Van Lenthe J. H.; Zwaans R.; Van Dam H. J. J.; Guest M. F. Starting SCF calculations by superposition of atomic densities. J. Comput. Chem. 2006, 27, 926–932. 10.1002/jcc.20393. 16557519

[ref47] Lehtola S. Fully numerical calculations on atoms with fractional occupations and range-separated exchange functionals. Phys. Rev. A 2020, 101, 012516 10.1103/PhysRevA.101.012516.

[ref48] Thomas N.; Smidt T.; Kearnes S.; Yang L.; Li L.; Kohlhoff K.; Riley P. Tensor field networks: Rotation- and translation-equivariant neural networks for 3D point clouds. arXiv 2018, arXiv:1802.08219 10.48550/arXiv.1802.08219.

[ref49] Plimpton S. Fast Parallel Algorithms for Short-Range Molecular Dynamics. Comp. Phys. 1995, 117, 1–19. 10.1006/jcph.1995.1039.

[ref50] Droghetti A.; Alfè D.; Sanvito S. Assessment of density functional theory for iron(II) molecules across the spin-crossover transition. J. Chem. Phys. 2012, 137, 124303 10.1063/1.4752411. 23020327

[ref51] Domingo A.; Àngels Carvajal M.; de Graaf C. Spin crossover in Fe(II) complexes: An ab initio study of ligand σ-donation. Int. J. Quantum Chem. 2010, 110, 331–337. 10.1002/qua.22105.

[ref52] Drautz R. Atomic cluster expansion for accurate and transferable interatomic potentials. Phys. Rev. B 2019, 99, 014104 10.1103/PhysRevB.99.014104.

[ref53] Shapeev A. V. Moment tensor potentials: A class of systematically improvable interatomic potentials. Multiscale Model. Simul. 2016, 14, 1153–1173. 10.1137/15M1054183.

[ref54] Domina M.; Patil U.; Cobelli M.; Sanvito S. Cluster expansion constructed over Jacobi-Legendre polynomials for accurate force fields. Phys. Rev. B 2023, 108, 094102 10.1103/PhysRevB.108.094102.

[ref55] NIST Computational Chemistry Comparisons and Benchmark Database, NIST Standard Reference Database, Number, Release 18; Johnson R. D., Ed.; NIST: Gaithersburg, MD, 2016.

[ref56] Nguyen V. H. A.; Lunghi A. Predicting tensorial molecular properties with equivariant machine learning models. Phys. Rev. B 2022, 105, 165131 10.1103/PhysRevB.105.165131.

