# Supplementary material for: Predicting the One-Particle Density Matrix with Machine Learning
Source: J Chem Theory Comput. 2024 May 31;20(11):4569–78. doi: 10.1021/acs.jctc.4c00042 (PMC11171273; doi:10.1021/acs.jctc.4c00042)
Supplement: Supplementary file 1 — ct4c00042_si_001.pdf [file ct4c00042_si_001.pdf]

Supplementary Information: Predicting The One-Particle Density Matrix With Machine Learning

S. Hazra, U. Patil, and S. Sanvito
School of Physics and CRANN Institute, Trinity College, Dublin 2, Ireland
(Dated: May 24, 2024)

I. CONVERGENCE ANALYSIS

In figure 1, we show the total energy (with respect to the ground-state energy) as a function of the iteration number, n, for S_2O computed with the DIIS [panel (a)] and SOS [panel (b)] mixing scheme. Furthermore, in panel (c) we present the norm of the difference between the ground state (converged) DM, ρ^{GS}, and that at the n-th iteration, ρ^n, also along the DIIS-driven SFC cycle.

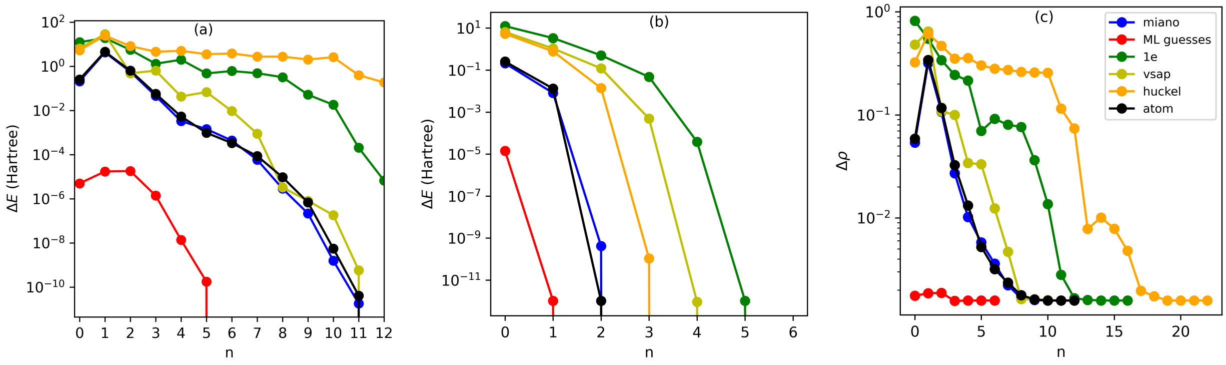

Figure 1: Analysis of the SCF cycle for S_2O. In panels (a) and (b) we show the total energy (measured with respect to the ground-state energy) as a function of the iteration number, n, for convergence driven by the DIIS and SOS mixing scheme, respectively. In panel (c) we present the norm of the difference between the ground-state converged DM, ρ^{GS}, and that computed at the n-th iteration, ρ^n. In this case we follow the DIIS-driven SCF cycle. For ease of visualization in all plots the y axis is on a logarithmic scale.

II. NON-SELF-CONSISTENT ENERGY AND FORCES

In figure 2 we present the parity plot diagram for the x [upper panel] and y [lower panel] component of the atomic forces acting on the H_2O atom lying in the x-y plane. These are computed for a set of 1000 distorted molecules obtained from the molecular dynamics trajectory used to generate the training set, but never used in the construction of the neural network. Since, the molecule are, by construction, always aligned in the x-y plane, there are no forces along z. The parity plot compares the fully converged DFT forces (y axis) with those predicted from the ML DM without operating any SCF iteration (x axis). Points on the parity line are predicted exactly. The graphs also show histograms of the distributions of the atomic forces.

In figure 3 we present the parity plot diagram for the energy of $[Fe(H_2O)_6]^{2+}$. Note that in this case we present energy and not forces, since we do not perform MD for $[Fe(H_2O)_6]^{2+}$, but we consider only rigid translation of the H_2O molecule with respect to the central Fe ion.

---

[1] P. Hohenberg and W. Kohn, *Inhomogeneous electron gas*, Phys. Rev. **136**, B864-B871 (1964).

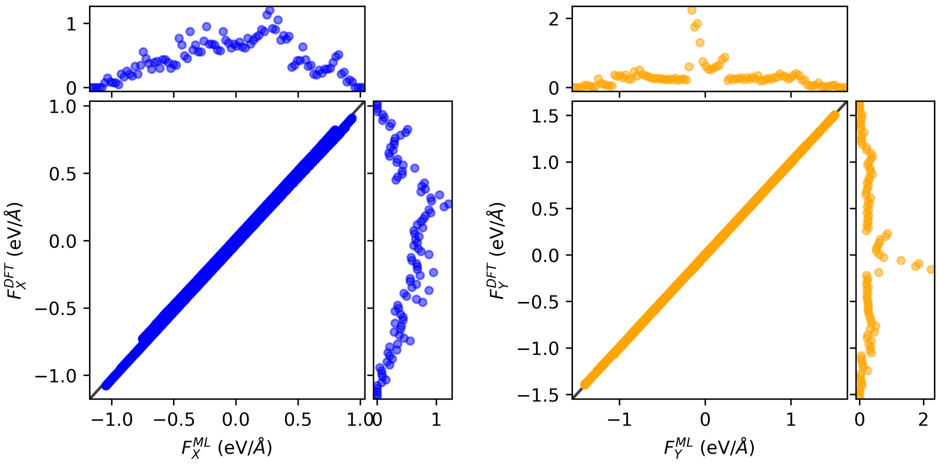

Figure 2: Parity plot for the $\alpha = x, y$ component of the atomic forces computed by using the ML DM, F_α^{ML}, with one SCF cycle, against the fully converged DFT ones, F_α^{DFT}. Data are here presented for a set of 1000 H_2O molecules extracted from the same molecular dynamics trajectory used to generate the training set. The upper panel is for the forces x component, while the lower panel is for the y component. The histograms on the side describes the frequency of the forces in the test set.

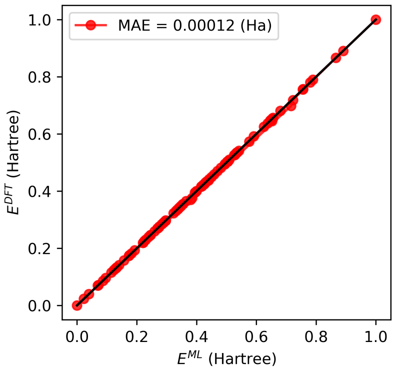

Figure 3: Energy parity plot for $[Fe(H_2O)_6]^{2+}$. On the x-axis we show the ground-state energy values obtained from the ML guess just after 1 SCF iteration. The fully converged DFT results are on the y-axis. Data are presented for 100 random structures taken from the test set. Note that, for $[Fe(H_2O)_6]^{2+}$, the energy values vary between $|1.72176 \times 10^3|$ to$|1.721630 \times 10^3|$ Ha, and for the ease of visualisation we shift the energies to be in the [0,1] Ha interval.
